# Post-Mortem Investigations for the Diagnosis of Sepsis: A Review of Literature

**DOI:** 10.3390/diagnostics10100849

**Published:** 2020-10-20

**Authors:** Chiara Stassi, Cristina Mondello, Gennaro Baldino, Elvira Ventura Spagnolo

**Affiliations:** 1 Legal Medicine Section—Department of Health Promotion, Maternal and Infant Care, Internal Medicine and Medical Specialties (PROMISE), University of Palermo, Via del Vespro 129, 90127 Palermo, Italy; chiara_stassi@libero.it (C.S.); gennarobld@hotmail.it (G.B.); 2 Department of Biomedical and Dental Sciences and Morphofunctional Imaging, University of Messina, Via Consolare Valeria 1, 98125 Messina, Italy; mondelloc@unime.it

**Keywords:** sepsis diagnosis, post mortem, morphology, histology, immunohistochemistry, biochemistry, microbiology

## Abstract

To date, sepsis is still one of the most important causes of death due to the difficulties concerning the achievement of a correct diagnosis. As well as in a clinical context, also in a medico-legal setting the diagnosis of sepsis can reveal challenging due to the unspecificity of the signs detected during autopsies, especially when no ante-mortem clinical data, laboratory, and cultural results are available. Thus, a systematic review of literature was performed to provide an overview of the main available and updated forensic tools for the post-mortem diagnosis of sepsis. Moreover, the aim of this review was to evaluate whether a marker or a combination of markers exist, specific enough to allow a correct and definite post-mortem diagnosis. The review was conducted searching in PubMed and Scopus databases, and using variable combinations of the keywords “post mortem sepsis diagnosis”, “macroscopic signs”, “morphology”, “histology”, “immunohistochemical markers”, “biochemical markers”, and “forensic microbiology”. The article selection was carried out following specific inclusion and exclusion criteria. A total of 44 works was identified, providing data on morphological aspects of the organs examined, histological findings, immunohistochemical and biochemical markers, and cultural assays. The review findings suggested that the post-mortem diagnosis of sepsis can be achieved by a combination of data obtained from macroscopic and microscopic analysis and microbial investigations, associated with the increased levels of at least two of three biochemical and/or immunohistochemical markers evaluated simultaneously on blood samples.

## 1. Introduction

Sepsis is a syndrome characterized by an excessive and dysregulated response to infections, often leading to multiorgan failure (MOF) and death, whose onset and evolution over time depend both on pathogen factors (microbial species and load, pathogenicity, virulence, etc.) and host factors (sex, age, genetic predisposition, comorbidities, immunosuppression, etc.) [1].

The septic process is the result of a systemic spread of the immune response starting from a primary infectious focus, most frequently lungs, abdominal cavity, urinary tract, soft tissues, and blood, and is usually ascribed to Gram-positive (mainly *S. aureus* and *S. pneumoniae*) and Gram-negative (mainly *E. coli*, *Klebsiella* sp., *Enterococcus* sp. and *P. aeruginosa*) bacteria; lately, an increase of sepsis cases sustained by fungal species was also observed [2].

Due to the difficulties in achieving a clinical diagnosis, sepsis remains one of the major causes of mortality, thus explaining the need for a prompt diagnosis and an appropriate treatment [1,3].

As well as in a clinical setting, a post-mortem diagnosis of sepsis is not always easy because macroscopic and histological findings, as well as the endogenous inflammation mediators produced, can in fact be found in other conditions, such as SIRS (Systemic Inflammatory Response Syndrome) or prolonged ischaemia [3,4,5]. In addition, the sole isolation of microbes from blood and/or tissue samples is not sufficient to diagnose sepsis as the cause of death, since it could reflect a non-pathological bacteraemia occurred ante-mortem, or a post-mortem translocation, or contamination [3,4,6]. For these reasons, several works have been produced in order to find specific markers for sepsis-related death, provided that a correct diagnosis cannot prescind from a comprehensive association of clinical history, autopsy, histological findings, and biochemical and microbiological results.

The following review provides an overview of the current methods and markers used, and their specificity, to perform a correct diagnosis of sepsis within a medico-legal context.

## 2. Methods

### 2.1. Search Strategy 

The works selected, screened independently by 4 researchers, included reviews, case reports, and prospective and retrospective case-control studies, all carried out between 2000 and 2019. The scientific articles chosen to realize the present review have been sorted from PubMed and Scopus databases. Based on the main approaches to date used for assessing a post-mortem diagnosis of sepsis, for this research, variable combinations of the keywords “post mortem sepsis diagnosis”, “macroscopic signs”, “morphology”, “histology”, “immunohistochemical markers”, “biochemical markers”, and “forensic microbiology” were used. The research on PubMed database produced 1770 articles and the research on Scopus database produced 302 articles. 

### 2.2. Study Selection

**Figure 1:**
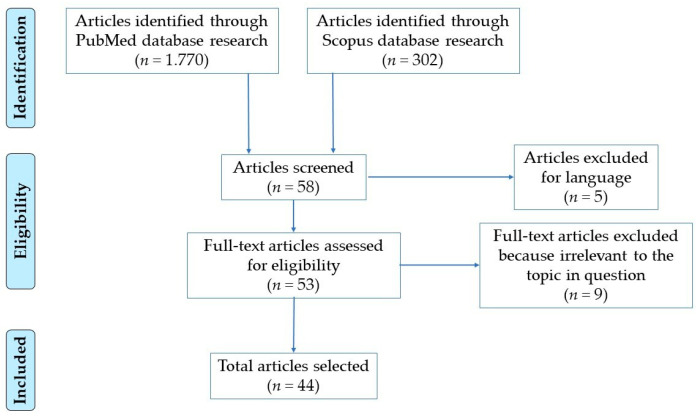
Research flowchart.

The eligible articles were chosen by 4 researchers independently according to the following inclusion criteria: English language; titles and abstracts suggesting a post-mortem analysis for the diagnosis of sepsis by morphological, histological, immunohistochemical, biochemical, and microbiological investigations. Then, the whole article was read if the abstract suggested it could potentially meet the inclusion criteria, and chosen if sepsis diagnosis was stated on the basis of the presence of a variable combination of SIRS criteria fulfilment, altered laboratory results, multiorgan dysfunction, alterations of acute phase mediators, identification of a septic focus, positive ante-, and/or post-mortem cultural assays. Articles in which the language was other than English or with titles, abstracts, and full texts irrelevant to the topic in question, were excluded. As a result, for the present work a total of 44 articles were selected from the PubMed database; no additional articles were selected from the Scopus database, since all the articles sorted coincided with those selected from the PubMed database (Figure 1). All works were further organized by discussed topic, author, publication date, study design, and main findings.

## 3. Results

### 3.1. Macroscopic and Histological Findings

The pathophysiological events underlying sepsis have a strong impact on the function of each organ [1,3,7] since the organ failure induced gives rise, in most cases, to morphological alterations that can be evaluated both macroscopically and microscopically. We start by suggesting some works describing the main post-mortem macroscopic and histological changes (Table 1) detected on septic organs [8,9,10,11,12,13,14,15,16,17,18,19,20,21].

With respect to the heart, macroscopic and histological changes, if detectable, are mostly unspecific. The changes described by Yang et al. [8] following autopsy investigation on 4 cases of *S. suis*-induced sepsis, included: petechiae and purpura of pericardium, myocardium and endocardium; myocardium degeneration; interstitial infiltrates of neutrophils and red blood cells. In their retrospective cohort study involving 235 patients hospitalized in an intensive care unit (ICU) and dead from sepsis or septic shock, Torgersen et al. [9] detected heart changes in just 53.6% of cases, which included myocardial ischaemia, acute ventricle dilation, and pericarditis. Sledzinska et al. [10] reported a case with just a focal contraction band necrosis of the heart. More specific septic-heart changes were described in three case reports from Rossi et al. [11], Sinicina et al. [12], and Maiese et al. [13], who detected atrial and ventricular areas of increased consistency and brown-greenish discoloration, with widespread intra- and extracellular Ca^2+^ deposition, interstitial perimysial fibrosis, myocytolytic myocytes, and inflammatory infiltrates mainly constituted by lymphocytes and multinucleated giant cells.

The unspecific changes usually detected in lungs were congestion, oedema, haemorrhages, embolism [8,9,10,11,13,14,15], and pleural effusion [15]. In a retrospective analysis on 3030 lung autopsy samples, de Matos Soeiro et al. [14], identified DAD (Diffuse Alveolar Damage) as the main histological finding (*n* = 1374/3030), noting that the corresponding alterations (extensive alveolar collapse; prominent hyaline membranes; obliterative fibrosis; marked septa neoformation) were significantly prevalent in those patients suffering from sepsis or liver cirrhosis (*p* < 0.0001). Since most of these patients were artificially ventilated, they also excluded a ventilator-induced DAD because the morphological pattern is mainly represented by thin hyaline membranes.

In an attempt to elucidate the pathogenic mechanisms underlying the onset of sepsis-AKI (Acute Kidney Injury), Aslan et al. [16] carried out histological investigations on kidney samples belonging to 27 patients, dead from sepsis-AKI, and 12 control patients with kidney cancer who underwent total nephrectomy. The main findings detected, reported on Table 1, were significantly prevalent in septic kidneys compared to controls (*p* < 0.0001). Since no significant alterations affected the glomeruli, except for a strong infiltrate of neutrophils and macrophages, the authors concluded that the tubulo-interstitial area, but not glomeruli, is mainly affected by the septic process, with a predominance of ATN (Acute Tubular Necrosis). These results were partly in contrast with those reported by Garofalo et al. [17], where unspecific changes like apoptosis, tubular cell vacuolization, swelling, and brush border injury were observed as the main findings (*n* = 996/1330), compared to the low prevalence of ATN (*n* = 236/1330).

Spleen changes were investigated by Gunia et al. [18], who performed a morphometric analysis on a total of 60 spleens, 30 of which obtained post mortem from patients dead from sepsis, the other 30 obtained from patients dead from cardiovascular non-infective diseases. Compared to controls, septic spleens showed lymphoid depletion of both B- and T-areas associated with a significant germinal center hyperplasia as signs of a reactive phenomenon (*p* < 0.001); in addition, in septic spleens, the B-area lymphoid depletion appeared more significant in cases of sepsis due to enterococcoemia compared to other Gram-negative species-induced sepsis (*p* < 0.001).

Brain alterations are not frequently detected, but the main findings described were oedema [8,9,10] and haemorrhages [17,19]. Other rare changes were reported by Sledzinska et al. [10], who found transtentorial herniation with cerebral oedema, and by Garofalo et al. [17], who described cases of central pontine myelinolysis and multifocal necrotizing leukoencephalopathy, together with evidence of microabscesses.

Reactive phenomena, centrilobular haemorrhagic necrosis, intrahepatic cholestasis, and acute cholangiolitis account for the main changes were detected in septic liver [8,9,17].

The most peculiar findings were associated to septic processes sustained by specific bacteria, such as those responsible for gas gangrene. An example is reported in a case report by Gioia et al. [20], where the septic process sustained by *Clostridium* spp. caused retroperitoneum and thigh muscles gas gangrene and iliopsoas myonecrosis, along with palpable crepitation and haemorrhagic-oedematous adipose tissues.

Finally, external signs may either be absent or vary significantly. As summarized by Rorat et al. [21], signs that can be detected, either on skin or mucosal surfaces, include abscesses, skin discolorations, rashes, or haemorrhagic lesions [8,19], which might testify to the development of a DIC (Disseminated Intravascular Coagulation) or the formation of septic embolisms. Sometimes, more specific lesions might help suggest the cause of sepsis, such as the presence of vesicular and erosive lesions in TEN (Toxic Epidermal Necrolysis) and SSSS (Staphylococcal Scalded Skin Syndrome), or cutaneous cellulitis (peau d’orange) and palpable crepitation in cases of gas gangrene [20].

### 3.2. Microbial Isolation

In both ante-mortem and post-mortem settings, cultural investigations are not sufficient, taken alone, to achieve a sepsis diagnosis; they have, however, a completing role when associated to other investigations since they can help (i) confirming an ante-mortem diagnosis, (ii) identifying the aetiological agent, or (iii) assessing the efficacy of eventual ante-mortem treatment [2,4,6].

Along with the autopsy investigations, in some of the above-mentioned works [8,10,15,19,20], as well as in other selected works further discussed [22,23,24,25,26], the authors also performed cultural assays in order to evaluate their usefulness in a post-mortem setting (Table 2).

The case reported by Abernathy et al. [19], concerning a 44-year-old woman who developed sepsis-induced MOF following the application of an intravenous device by inexperienced personnel, is an example of the confirmative role of post- mortem cultural investigations when ante-mortem cultural data were available. Specifically, cultural assays carried out either on blood obtained ante mortem and on pericardial fluid, intravenous tubing and saline bag obtained post mortem, confirmed a Methicillin-Resistant *Staphylococcus aureus* (MRSA) as the causative agent of sepsis.

The role of the aetiological agents of sepsis in the post-mortem analysis was well established in several works. Yang et al. [8], for example, isolated *Streptococcus suis* type 2 from blood and other tissue samples in 3 of the 4 cases reported, who were all farmers referring they had eaten pork from ill pigs the day prior the development of sepsis, while Sledzinska et al. [10], combining cultural assays and PCR (Polymerase Chain Reaction) technique for molecular analyses, identified Dr ^+^ *E. coli* as the aetiological agent of gestational interstitial tubulonephritis and chronic pyelitis-induced sepsis in a 23-year-old pregnant woman.

Suzuki et al. [15] isolated *Clostridium* spp. from the blood of a 67-year-old woman who developed a PIC (Pneumatosis Cystoides Intestinalis)-induced sepsis, while in Gioia et al.’s work [20], *Clostridium perfringens*, isolated in the aortic blood of a 60-year-old woman, was identified as the causative agent of the gas gangrene developed by the patient the day after a polypectomy procedure.

Ploy et al. [22] made an aetiological diagnosis of sepsis-induced purpura fulminans in a child following the identification of *Neisseria meningitidis* serogroup C by cultural and PCR assays carried out on blood, urine, CSF(cerebro-spinal fluid), throat swabs, purpuric lesions, and adrenal gland samples obtained post mortem.

Kawaguchi et al. [23] reported the case of a sudden unexpected death of a neonate 6 days after birth. The autopsy investigation revealed several bacterial foci in lungs and intracerebral vessels, while microbial cultures on post-mortem blood and faeces samples revealed the presence of *Streptococcus agalactiae*, leading the authors to diagnose a late onset group B streptococcal sepsis as the cause of death.

Frati et al. [24] reported the case of a woman who died from sepsis following the transfusion of seven bags of red blood cells due to a haemorrhagic shock in the early post-partum period. The isolation of *Y. enterocolitica* on post-mortem blood samples raised the suspect of a post-transfusion sepsis, which was further confirmed by the detection of *Y. enterocolitica* serotype O:9 antibodies in the plasma of one of the donors.

Sarvari et al. [25] presented the case of a 76-year-old woman admitted to the hospital with abdominal pain, vomit, MOF signs, and laboratory results suggestive of septicemia. Even though the exact source of the infection could not be identified, both the severe gastrointestinal symptoms and the abdominal CT scans, together with the isolation of *Clostridium perfringens* in cultures carried out on intestinal and subcutaneous tissue of the chest, suggested a diagnosis of emphysematous gastritis, a rare disease characterized by the invasion of the stomach wall by gas-producing bacteria.

Finally, the work from D’Ovidio et al. [26] is an example of the role of microbial investigations in assessing the efficacy of antibiotic treatments. The authors described the case of a 29-year-old male with systemic lupus erythematosus who developed sepsis following an incision of a gluteal abscess. Even though he was subjected to a strong antibiotic therapy, his conditions kept worsening and he died from MOF and cardiopulmonary arrest. Ante-mortem blood cultures always resulted negative; on the other hand, the post-mortem cultural assays performed on cardiac tissue and valves, liver, CSF, and blood samples allowed the isolation of MDR *K. pneumoniae* as the aetiological agent of the septic process, also explaining the inefficacy of the strong antibiotic treatment.

### 3.3. Immunohistochemical Markers

Several immunohistochemical markers to date were tested as possible post-mortem markers for the diagnosis of sepsis [3,4,12,14,21,27,28,29]. The lung, representing the mostly impaired organ in sepsis, has rapidly become the main target for immunohistochemical assays in order not only to improve the knowledge of sepsis pathophysiology, but also to establish whether an immunohistochemical marker exists, specific enough to allow a differential diagnosis between sepsis and non-sepsis fatalities within a medico-legal context [30,31,32,33,34,35,36,37,38,39,40,41] (Table 3).

Possible immunohistochemical markers of sepsis were investigated post mortem in four works from Tsokos et al. [30,31,32,33], and in each study the immunoreactivity was tested both in sepsis and non-sepsis control cases. E-selectin [30] was tested on lung samples obtained from 37 cadavers, 6 of which dead from sepsis, 7 from possible sepsis-associated fatalities, and 24 from non-sepsis fatalities. E-selectin (Endothelial-Selectin) immunoreactivity appeared significantly higher in all sepsis cases compared to possible sepsis-associated fatalities and non-sepsis cases (*p* < 0.05). VLA-4 (Very Late Activation antigen 4, or CD49d/CD29) and ICAM-1 (Intercellular Adhesion Molecule 1) were assessed on lung samples obtained post mortem from 8 sepsis cases and 22 non-sepsis control cases [31]. All sepsis cases showed a strong VLA-4 expression in intravascular, interstitial and intra-alveolar pulmonary leukocytes, unlike the non-sepsis control cases where immunoreactivity was weak in interstitial leukocytes and absent in intravascular and intra-alveolar leukocytes; ICAM-1 was strongly expressed in endothelial cells of pulmonary vessels, pulmonary macrophages, and lymphocytes in all sepsis but not in the non-sepsis control cases. For both molecules, the immunoreactivity differences between the two groups resulted as being statistically significant (*p* < 0.001). Leukocytic immunoreactivity of lactoferrin (LF) and lysozyme (LZ) was investigated on lung samples obtained from autopsy cases of sepsis-related (*n* = 13) and non-sepsis-related fatalities (*n* = 14) [32]. LF expression resulted as being significantly higher in sepsis cases compared to non-sepsis control cases (*p* < 0.001), unlike LZ for which no immunoreactivity differences were observed between the two groups (*p* < 0.152). Finally, the expression of VEGF (Vascular Endothelial Growth Factor) was assessed in lung samples obtained from 18 cadavers, 8 of which dead from sepsis and 10 dead from other natural and unnatural causes [33]. A strong immunoreactivity was detected in samples from all control cases, while negative results were observed in sepsis cases, with a significant difference between the two groups (*p* < 0.001). The authors postulated that the VEGF decrease in sepsis might be due either to the release of bacterial endotoxins or to a down-regulation of VEGF receptors following the systemic inflammatory response onset during sepsis.

Lung samples from a sepsis group (*n* = 9) and from a non-sepsis control group (*n* = 8) were also tested by Miyashita et al. [34] for TNF-α (Tumor Necrosis Factori-α) immunoreactivity, which resulted as being significantly higher in sepsis compared to non-sepsis control cases (*p* < 0.05).

The expression of VEC (Vascular Endothelial-Cadherin), a Ca^2+^ -dependent endothelial cell–cell adhesion molecule, was investigated on lung samples by both Muller et al. [35] and Hervig et al. [36]: in the first work, immunoreactivity was tested in a control group of 20 non-sepsis patients and a group of 19 patients who died from Gram-negative sepsis; in the second work, 20 cases were patients with acute respiratory distress syndrome (ARDS) who died from Gram-negative sepsis, while 41 were control patients affected by a malignant lung tumor. In both works, the authors found a significant expression of VE-cadherin in control cases compared to the weak immunoreactivity in sepsis cases (*p* < 0.05). The same immunoreactivity pattern was reported by Muller et al. [35] for ACE (Angiotensin Converting Enzyme), a multifunctional molecule that regulates blood pressure, coagulation, cell proliferation, bradykinin, and other kinins degradation.

An et al. [37] carried out their immunohistochemical analysis on monocytes/macrophages chemokine receptor CCR2 (C-C Motif Chemokine Receptor 2) and CX3CR1 (C-X3-C Motif Chemokine Receptor 1), both G protein-coupled transmembrane receptors with chemoattractant function. The immunoreactivity of both molecules was tested on lung samples obtained from 8 non-sepsis control cases and 9 sepsis cases: both CCR2^+^ and CX3CR1^+^ mononuclear cells were significantly higher in the sepsis group compared to controls (*p* < 0.01).

Procalcitonin (PCT) is a well-known marker of sepsis, whose levels are routinely evaluated in biological fluids both in clinical and autopsy settings, and whose role is that of differentiating bacterial from non-bacterial sepsis. In their work, Maiese et al. [38] suggested PCT as a possible immunohistochemical marker for sepsis, and tested its expression on brain, heart, lung, liver, and kidney samples from 10 sepsis cases and 5 non-sepsis control cases. The study revealed PCT negativity on samples belonging to the non-sepsis group; on the other hand, the sepsis group showed PCT immunopositivity with differences in localization, being expressed in blood vessels and cytoplasm of brain, heart, and lung tissue in the hepatocytes; and in glomeruli, renal tubules, and blood vessels of kidney.

The few studies about the structural alterations of cardiac cells in a septic context, led Ventura Spagnolo et al. [39] to search for immunohistochemical markers for septic cardiomyopathy. Specifically, the authors focused on the potential of the sarcoglycan sub-complex, a member of the dystrophin-glycoprotein complex (DGC), which plays a key role in sarcolemmal stabilization. To this end, a retrospective observational study was performed on forensic autopsies carried out on 10 sepsis cases and 10 non-sepsis control cases. The immunohistochemical analysis showed a significant decrease of sarcoglycan positivity in sepsis cases compared to control cases (*p* < 0.001), suggesting that the reduction in sarcoglycan expression could contribute to plasma membrane damage and increased sarcolemmal permeability in a septic context.

In Galassi et al. [40], the immunoreactivity of α-SMA (α-Smooth Muscle Actin), fibronectin, MMP-9 (Matrix Metallopeptidase 9), ICAM-1, caspase-3, lactoferrin (LF), and CD15 was tested on heart samples from a control group of 25 non-sepsis cases and from a sepsis group of 56 cases in order to find out if one of them could be used as a specific marker of myocardial dysfunction in sepsis. The immunohistochemical assays showed no differences of immunoreactivity for α-SMA, fibronectin, MMP-9, caspase 3, and CD54 between the two groups. Conversely, significant differences were observed for both LF and CD15, resulting as positive in 33/56 sepsis cases, a proportion that results significantly higher than controls.

Maiese et al. [41] tested TREM-1 (Triggering Receptor Expressed on Myeloid cells-1) immunoreactivity on brain, heart, lung, liver, and kidney samples from 28 sepsis cases. Immunopositivity was detected in pulmonary blood vessels and cytoplasm of myelomonocytes, in heart blood vessels, in hepatocytes, and in glomeruli, renal tubules and kidney blood vessels. Finally, anti-TREM-1 antibody immunopositivity was not detected in samples belonging to subjects who died from non-infective causes (control group).

### 3.4. Biochemical Markers

The systemic inflammatory response related to sepsis is characterized by a massive release in the blood compartment of several pro-inflammatory mediators, some of which are to date routinely investigated in living patients, being suggestive of a septic condition [3]. As an integral part of sepsis pathophysiology, many authors asked whether these mediators could be used as post-mortem markers of sepsis [3,42]. Unfortunately, not every mediator is suitable for this kind of investigation since several factors may interfere with a correct interpretation of the results (time elapsed from death, possible supravital reactions, leakage from cell deterioration, blood haemolysis, diffusion/redistribution dependent on concentration gradients, analytical procedures). For this reason, post-mortem biochemical investigations are limited to those few markers that are relatively stable in peripheral blood and some body fluids [5,21,43]. The following studies [44,45,46,47,48,49,50,51,52,53,54,55,56] provide an up-to-date overview of the molecules assessed as potential post-mortem biochemical markers of sepsis (Table 4).

Tsokos et al. [44] assessed the levels of IL-6 and CRP (C-Reactive Protein) on blood samples obtained from 8 sepsis cases and 16 non-sepsis control cases. Both molecules were considered good markers for the post mortem diagnosis of sepsis since the levels detected were significantly higher in sepsis than in non-sepsis control cases. In addition, an increase in IL-6 levels and a decrease in CRP levels were detected with increasing PMI (post-mortem interval) in sepsis cases.

Reichelt et al. [45] evaluated the levels of three inflammatory mediators, IL-1β, sIL-2R, and LBP (Lipopolysaccharide Binding Protein), all playing a key role in the host systemic inflammatory response. Measurements were carried out on blood samples obtained during the early post-mortem interval from 8 sepsis cases and 16 non-sepsis control cases; blood samples were also obtained ante mortem from the patients of the sepsis group. No statistically significant differences were observed in IL-1β levels between sepsis and non-sepsis control cases; conversely, both sIL-2R and LBP levels revealed elevated in all sepsis cases, both ante mortem and post mortem, but not in any control cases, thus inducing the authors to consider both markers suitable for a post-mortem diagnosis of sepsis (*p* < 0.01).

Five molecules, PCT (Procalcitonin), CRP, TNF-α, IL-6, and IL-8, were tested by Schrag et al. [46] in blood samples obtained from 8 sepsis cases and 10 non-sepsis control cases. Significant differences between the two groups were only observed for CRP and PCT levels (respectively, *p* < 0.001 and *p* < 0.016), which were considered reliable post-mortem markers of sepsis. CRP and PCT levels were also assessed in vitreous humor and cerebrospinal fluid from the same groups (data not shown), reporting only vitreous humor PCT levels significantly higher in sepsis than in non-sepsis control cases. The same authors [47] further evaluated the presence of PCT and CRP in pericardial fluid, and compared the levels observed to those found in femoral blood samples in order to establish whether pericardial fluid could be considered a valuable measurement matrix when blood samples are not available. To this end, CRP and PCT levels were investigated in blood and pericardial fluid samples obtained post mortem in 12 sepsis cases and 28 non-sepsis control cases (results showed in Table 4). Unlike CRP, PCT levels assessed post mortem in pericardial fluid showed ranges similar to those found in blood samples, both in sepsis and control cases. The authors concluded that PCT, but not CRP, measurement in pericardial fluid obtained post mortem can be considered a possible alternative to blood, with a 90% sensibility and an 89.29% specificity.

With the same aim, Schrag et al. [47] and Augsburger et al. [48] measured PCT and LBP levels in blood and pericardial fluid samples obtained post mortem from 12 sepsis cases and 30 non-sepsis control cases. The authors concluded that both blood PCT and LBP are accurate post-mortem biomarkers for the diagnosis of sepsis-related fatalities. Measurements carried out on pericardial fluid showed that PCT levels were significantly higher in sepsis than in non-sepsis control cases; moreover, increases in blood and pericardial fluid PCT levels corresponded in 9/12 sepsis cases, while blood and pericardial fluid PCT levels below the detection limit corresponded in 100% of the control cases. Conversely, they highlighted that LBP measurement in pericardial fluid cannot be considered as an alternative option to blood, because blood and pericardial fluid LBP levels were incomparable in both sepsis and non-sepsis cases.

Palmiere et al. engaged in a series of works aimed to assess the value of several molecules as post-mortem markers of sepsis. In the first one [49], sTREM-1, PCT, and CRP levels were measured in blood samples obtained post mortem from 16 sepsis cases and 16 non-sepsis control cases. The comparison of the levels of these three markers revealed that, taken individually, all of them allowed for sepsis-related death diagnosis with comparable sensibility; moreover, in all sepsis cases no normal levels of the three markers were observed simultaneously, but in each sepsis case, at least two parameters were increased. In most samples of the control group, sTREM-1, PCT, and CRP levels were normal. On the basis of the obtained results, the authors concluded that, taken alone, sTREM-1 is not better than PCT or CRP as a marker for the diagnosis of sepsis in the forensic setting. sTREM-1 levels were also assessed in pericardial fluid and urine obtained post mortem (data not shown), and then compared to serum levels, in order to evaluate their usefulness as an alternative measurement matrices: the comparative analysis showed that pericardial fluid, but not urine, can be a valid alternative when blood samples are not sufficient or unavailable. In another work [50], the sCD14-ST (soluble Cluster of Differentiation 14-Subtype) was tested as a post-mortem marker of sepsis in blood and pericardial fluid obtained from 19 sepsis cases and 18 non-sepsis control cases. Compared to PCT and CRP, sCD14-ST showed high sensitivity (94.74%), but low specificity (44.44%). The analysis carried out on couples of markers (PCT and sCD14-ST; PCT and CRP; CRP and sCD14-ST) and on the three markers together, resulted in increased levels in almost every sepsis case and normal values in almost every control case (data not shown). Finally, no correspondence was detected between blood and pericardial fluid levels of sCD14-ST in either sepsis or control groups, thus indicating that pericardial fluid cannot be considered an alternative matrix to blood for sCD14-ST measurement. Other molecules were tested by the same authors, and compared to PCT and CRP levels. sTREM-1 and sIL-2R levels were assessed in blood, pericardial, and pleural fluids obtained post mortem from 12 sepsis cases and 20 control cases [51]: the levels of each tested marker resulted in being significantly higher in blood, pericardial, and pleural fluids from sepsis than non-sepsis control cases (*p* < 0.001), leading the authors to also consider pleural fluid a valid alternative matrix when blood is not available. Copeptin levels were evaluated in blood samples obtained post mortem from 28 sepsis cases and 28 non-sepsis control cases [52]. Analytical investigations showed not only that copeptin levels were significantly higher in sepsis cases compared to non-sepsis ones (*p* < 0.001), but also that they correlated with PCT, CRP, and IL-6 blood levels. Endocan levels, compared to PCT and CRP levels, were measured in blood and pericardial fluid samples obtained post mortem from 16 sepsis cases and 16 non-sepsis control cases [53]. Blood levels of endocan resulted as being significantly higher in the sepsis group, and almost undetectable in 11/16 control cases (*p* < 0.001), while no significant differences were detected in endocan levels in the pericardial fluid of both sepsis and non-sepsis control groups. Hence, endocan can be considered a suitable marker for the diagnosis of sepsis-related deaths when measured in serum, but not in pericardial fluid samples. More recently, the same study group has assessed a novel molecule, the pancreatic stone protein/regenerating protein [54], of which increased blood levels have been detected in living septic patients. Its levels were evaluated, as usual, in blood samples obtained post mortem from cadavers divided in sepsis-related deaths group, local infections group, and non-sepsis ICU patients group, and compared to PCT, CRP, IL-6, and sTREM-1 levels. Pancreatic stone protein/regenerating protein levels were revealed as being significantly higher in sepsis than in control cases (*p* < 0.001), and a significant positive correlation was observed between this molecule and PCT in sepsis cases. On the basis of these results, the authors concluded that a pancreatic stone protein/regenerating protein can be used as a post-mortem marker for the diagnosis of sepsis.

Tettamanti et al. [55] measured NT-proBNP, troponin T, and troponin I levels in sepsis cases in order to assess whether an increase of these biomarkers correlates to macroscopic or microscopic signs of myocardial damage or cardiac dysfunction. To this end, NT-proBNP, troponin T, and troponin I (together with PCT and CRP) levels were evaluated on blood samples obtained post mortem from 16 sepsis cases and 16 non-sepsis control cases. The results revealed that troponin I, troponin T, and NT-proBNP levels, compared to controls, increased in sepsis-related deaths even if coronary artery disease, myocardial ischaemia, or signs of heart failure were absent. In addition, a significant correlation was found when the markers levels were compared in couples (NT-proBNP/Troponin I and NT-proBNP/Troponin T—*p* < 0.05 in each case).

In their work, Unuma et al. [56] tested the PSEP (Presepsin). The authors measured the levels of PSEP, CRP, and PCT in blood samples obtained from 19 sepsis cases and 74 non-sepsis control cases, and the comparison of the values observed showed that, compared to CRP and PCT, PSEP is even a superior biomarker for the post-mortem diagnosis of sepsis (*p* < 0.01).

## 4. Discussion

According to the Third International Consensus Definitions (Sepsis-3), sepsis is defined as a “*life-threatening organ dysfunction caused by a dysregulated host response to infection*”, which can result in multiorgan failure and death due to a combination of tissue hypoperfusion, impairment of the coagulation system, and oxidative stress [1]. Within a clinical setting, a correct diagnosis of sepsis requires the simultaneous presence of three main features: (1) a primary infectious focus; (2) ≥2 SIRS criteria (T > 38 °C or < 36 °C; heart rate > 90/min; respiratory rate > 20/min or PaCO_2_ < 32 mmHg; WBC count > 12.000/mm^3^ or < 4.000/mm^3^); (3) acute organ dysfunction, documented by a SOFA Score ≥ 2 points [1,3]. Nonetheless, the diagnostic process is not as easy as it may seem either because these features are seldom present together, or because SIRS and organ dysfunction—taken alone—are not specific aspects of sepsis, but may be present in many other pathological conditions; SIRS in particular, can just be the result of the host response to a general exogenous insult, be it infectious or not [1].

Sepsis diagnosis may be challenging also in a medico-legal setting, especially if clinical data or ante-mortem laboratory and cultural results are not available. In this context, proper investigations may either help a post-mortem diagnosis of sepsis when the cause of death is unknown, or confirm an ante-mortem suspect [3,4,5,57,58]. Moreover, a reliable post-mortem diagnosis is of utmost importance for the medico-legal implications related to the identification of professional liability when sepsis-related death is ascribed to malpractice (e.g., nosocomial infections, mismanagement of surgical wounds or specific infections, inadequate asepsis procedures, low hygienic standards, inappropriate therapies, etc.), or identification of sepsis as cause of death in patients with infectious complications arising from different conditions (e.g., burns, immobilization, alcoholism, intravenous drug addiction, big traumas, food intoxication, etc.) [4,59,60,61,62,63]. It is thus fundamental to provide a complete forensic analysis of the case in order to perform a certainty diagnosis. To this end, the present review summarizes the main investigations that could be implemented in a post-mortem assessment.

The first aspect is to investigate are the morphological changes of the organs, resulting from the effect of the pathophysiological events underlying sepsis (hypotension and ischaemic sufferance of internal organs; systemic inflammatory response; direct tissue damage; DIC; platelet aggregation impairment; production of anaphylactoid mediators—e.g., C5a) [1,2,3]. At external inspection, only rarely skin lesions/discolorations might address towards a specific infection [20,21]; at internal examination, many organs usually appear congested, or haemorrhagic, and may present infectious foci/abscesses, while fluid effusions or haemorrhages can be evaluated in serous membranes. The histological investigation might reveal variable leukocytic infiltrates, vascular thrombi, fibrosis, tissue damage signs, even microbial infiltrates [8,9,10,11,12,13,14,15,16,17,18,19,20,21]. Unfortunately, none of these signs are sensitive or specific, since each one reflects the effects of either a generic inflammatory insult, be it infectious or not, or a haemodynamic impairment of the organ function. Therefore, each pathological condition associated with systemic inflammation can generate the same organ alterations, providing no specific macroscopic and microscopic findings [1,2,3]. A more specific finding, represented by calcification foci, was only detected on heart samples from long-term severe sepsis cases in just three works [11,12,13]. In an attempt to make a differential diagnosis with other pathological conditions, Rossi et al. [11] excluded a possible association of these findings either with idiopathic giant cell myocarditis or calcified myocardial necrosis following infectious myocarditis, since in both cases, calcification is very uncommon and the way inflammatory mediators infiltrate and their infiltrate composition differ significantly. Subsequently, macroscopic signs and histological findings cannot be considered, taken alone, suitable markers for a post-mortem diagnosis of sepsis.

In order to fill the gap left by routine macroscopic and microscopic analyses, other *post-mortem* investigations were proposed. Among these, cultural investigations carried out on blood and tissue samples were suggested for their crucial role in the achievement of the sepsis diagnosis, though a major concern was related to the correct interpretation of the results. Bacterial growth can in fact correspond not only to a genuine positive (pure growth of a specific pathogen colonizing an otherwise sterile organ or fluid), but also to post-mortem translocation (bacterial migration from the mucosal surface into the blood and internal organs after death) or contamination (incidental introduction of bacteria into the samples when they are obtained using non-sterile tools or operating in non-sterile environments) [2,3,4,6]. Post-mortem translocation can be easily avoided if samples are obtained within 24 h from death, or if the body is correctly stored at 4 degrees Celsius before autopsy, while contamination can be reduced to levels very close to those found in samples obtained in life (four to six percent) if stringent precautions are taken [6,64]. Based on our research, in the context of a post-mortem diagnosis of sepsis, cultural investigations can be considered reliable only if supported by other investigations and clinical reports, and can only contribute to the definition of the aetiological agent causing sepsis or to the corroboration of a sepsis suspect [8,10,13,15,18,19,20,22,23,24,25,26].

The immunohistochemical and biochemical approaches were also suggested on the basis of the knowledge of the events regulating the signal cascades in sepsis [3,4,5,7,8,11,13,18,24,25,26,39,40].

Immunohistochemistry is a useful, simple, and repeatable post-mortem investigation providing effective findings for the assessment of several causes of death [65,66,67]. The spectrum of the molecules immunohistochemically tested for sepsis is wide, and includes cell–cell adhesion molecules, chemoattractant molecules, acute phase mediators, growth factors involved in angiogenesis, and pro-inflammatory cytokines. In order to assess their value as post-mortem biomarkers of sepsis, each of them was tested in samples obtained from subjects dead both from sepsis or non-sepsis causes. Immunohistochemical markers considered suitable for this purpose include: E-selectin [30], VLA-4, and ICAM-1 (CD54) [31], all adhesion molecules involved in leukocytic attraction and migration through the endothelial barrier towards the inflammatory compartment; lactoferrin (LF) [32], an iron-binding protein whose role is to take iron from bacteria in order to limit their growth; TNF-α [34], one of the major pro-inflammatory mediators; CCR2 and CX3CR1 [37], involved in leukocytic chemotaxis; PCT [38], whose expression is up-regulated during sepsis; CD15 [40] and TREM-1 [41]. Other suitable immunohistochemical markers characterized by the decrease of their expression in sepsis cases compared to controls, include: VEGF [33], involved in neoangiogenesis and vascular permeability; VEC [35,36], a cell–cell adhesion molecule involved in the endothelial barrier remodeling in response to an inflammatory insult; ACE [35], which is also involved in the modulation of the bradykinin system; sarcoglycan [39], involved in sarcolemmal stabilization, whose levels decrease in septic heart. Since no significant immunoreactivity differences were detected between sepsis and non-sepsis cases, lysozyme (LZ) is the only marker tested, which was not considered suitable for a post-mortem diagnosis of sepsis.

As for the biochemical markers, the most investigated in literature for a sepsis diagnosis were PCT and CRP [44,46]: the first one is an acute phase mediator whose increase is observed in all conditions characterized by an inflammatory response, while PCT is a more specific marker for bacterial sepsis. The levels of both molecules are routinely assessed within a clinical setting given their positive correlation with the severity of the pathological condition and with treatment response; such positive correlation was also observed in a post-mortem setting, and PCT in particular proved to be a good marker when measured both in blood and pericardial fluid samples [47], unlike CRP, whose potential as a post-mortem marker is related to blood, but not pericardial fluid, measurement. Other tested molecules considered suitable markers when tested on blood samples include [44,45,46,47,48,49,50,51,52,53,54,55,56]: sIL-2R; LBP; sTREM-1; presepsin; copeptin; endocan; troponins I, and T, together with NT-proBNP; pancreatic stone protein/regenerating protein. A few studies also tested IL-6, but the contrasting results obtained did not allow to confirm its usefulness as a post-mortem marker [44,46]. Pericardial fluid has proven a good alternative matrix to blood for the measurement of some of these molecules, including PCT [47,48], sTREM-1 [49,51], and sIL-2R [51], whose levels revealed comparable to the levels observed in blood samples, with significant differences between sepsis and non-sepsis control cases. On the contrary, LBP [48], endocan [53], and presepsin [50,56], were not described useful when investigated in pericardial fluid samples.

The above discussed arguments on postmortem diagnosis in sepsis-related death acquire great importance in the context of COVID-19, a severe disease due to Sars-CoV-2 infection, which spread across the world, bringing the World Health Organization (WHO) to declare a pandemic [68,69,70]. In fact, several pieces of research highlighted the importance of the post-mortem infection assessment using macroscopic and microscopic autopsy findings, immunohistochemistry for inflammatory markers, and PCR analysis for virus detection [71,72]. 

## 5. Conclusions

**Figure 2:**
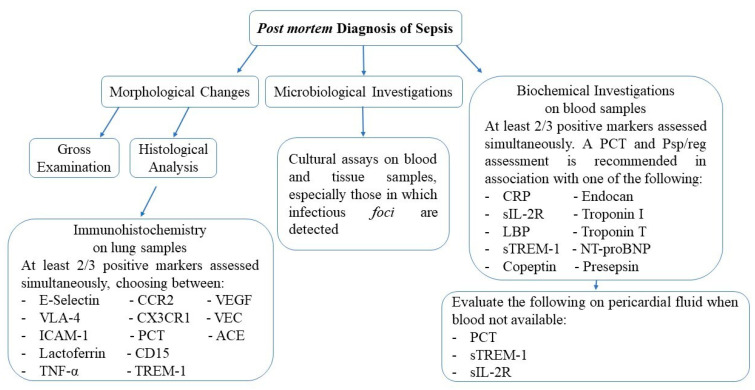
Schematic overview of the main investigations needed for a *post-mortem* sepsis diagnosis, with indications for a possible, effective, procedural combination.

The achievement of a correct diagnosis of sepsis is challenging both in living patients and after death, due to the fact that the typical features of this pathological condition (systemic inflammatory response, clinical signs, internal organs changes) are not always present simultaneously and, taken alone, can relate to many other diseases. Based on the systematic review of the works discussed, we can thus conclude that, in a forensic context, a sepsis diagnosis results from a comparative analysis of multiple features. A post-mortem diagnosis of sepsis is usually facilitated when clinical records and ante-mortem laboratory/cultural investigations are available: in such a case, suggestive macroscopic and histological findings, together with cultural investigations matching the ante-mortem ones, are sufficient enough to confirm the diagnosis. On the contrary, when no or partial ante-mortem data are available, a post-mortem diagnosis of sepsis can be achieved, with reasonable confidence, by means of a comparison of the features discussed in the present work. A possible effective combination can be expected when positive microbial cultures (especially when carried out on samples from infectious foci, when detected at autopsy) are associated to increased levels of at least two of three biochemical and/or immunohistochemical markers evaluated simultaneously on blood samples, relying on pericardial fluid when blood is not available or not sufficient; given its well established role as a bacterial sepsis biomarker and its positive correlation with sepsis when measured both on blood and pericardial fluid, we also suggest the inclusion of PCT among the markers chosen (Figure 2).

## Figures and Tables

**Table 1:** Main sepsis-related macroscopic and histologic findings observed post mortem.

**Reference**	**Yang et al.** [8] **(2009)** **Case Series**	**Torgersen et al.** [9] **(2009)** **Retrospective Study**		**Sledzinska et al.** [10] **(2010)** **Case Report**
**Findings**	**External Inspection** - Petechiae (*n* = 4/4). **Serous Cavities** - Hydrothorax (*n* = 4/4); - Hydropericardium (*n* = 3/4); - Ascites (*n* = 1/4). **Heart** - Petechiae and purpura of pericardium, myocardium, and endocardium (*n* = 4/4); - Myocardium degeneration (*n* = 4/4); - Interstitial neutrophils and red blood cells infiltration (*n* = 4/4). **Lungs** - Congestion (*n* = 4/4); - Oedema (*n* = 4/4); - Haemorrhage (*n* = 4/4); - Neutrophils infiltration (*n* = 4/4); - Capillary microthrombi (*n* = 4/4). **Kidneys** - Congestion (*n* = 4/4); - Glomerular capillaries microthrombi (*n* = 4/4); - Tubular cells degeneration (*n* = 4/4); - Multi-spot necrosis with neutrophils infiltration (*n* = 4/4); - Hyaline casts (*n* = 4/4). **Spleen** - Decreased size (*n* = 4/4); - Atrophic white pulp and expanded red pulp with neutrophils infiltration (*n* = 4/4). **Liver** Focal and spotty necrosis (*n* = 4/4). **Adrenal glands** Widespread haemorrhage (*n* = 4/4). **Brain** Mild cerebral oedema with neuron degeneration, neuronophagia, and satellite phenomenon (*n* = 4/4).	**Heart** - Myocardial ischaemia (*n* = 83/235); - Acute ventricle dilation (*n* = 27/235); - Pericarditis (*n* = 21/235). **Lungs** - Oedema (*n* = 137/235); - Pneumonia (*n* = 97/235); - Pleural effusions (*n* = 47/235); - Tracheo-bronchitis (*n* = 68/235); - Embolism (*n* = 30/235). **Liver** - Steatosis (*n* = 78/235); - Cholestasis (*n* = 33/235); - Hypoxic liver damage (*n* = 31/235). **Gastrointestinal Tract** - Chronic peritonitis (*n* = 55/235); - Mesenteric ischemia (*n* = 42/235). **Spleen** Enlargement and softening (n = 68/235). **Pancreas** Necrotizing pancreatitis (*n* = 20/235). **Kidneys and Urinary Tract** - Cystitis (*n* = 83/235); - Kidney swelling (*n* = 53/235); - Kidney ischemia (*n* = 27/235). **Brain** Oedema (*n*= 32/235).		**Kidneys and Urinary Tract** Macroscopic findings: - Hydronephrosis; - Acute pyelonephritis; - Abscesses. Histologic findings: - Tubulo-interstitial nephritis with microabscesses; - Chronic suppurative pyelitis. **Lungs** Macroscopic findings: - Congestion and oedema. Histologic findings: - Microvascular embolisms from syncytiotrophoblasts and megacaryocytes; - Focal intra-alveolar haemorrhages. **Heart** Focal contraction band necrosis. **Brain** - Congestion and oedema; - Transtentorial herniation. **Spleen** - Congestion; - Neutrophils infiltration.
**Reference**	**Rossi et al.** [11] **(2003)** **Case Report**	**Sinicina et al. [12]** **(2005)** **Case Report**		**Maiese et al. [13]** **(2019)** **Case Report**
**Findings**	**Heart** Macroscopic findings: - Global dilation with flaccid ventricular walls; - Interstitial fibrosis with chalky whitish punctate foci. Histologic findings: - Widespread interstitial perimysial fibrosis; - Myocytolytic myocytes foci; - Calcified myocardial cells, mixed with macrophages, T lymphocytes, and multinucleated giant cells. **Lungs** Congestion. **Liver** Congestion. **Spleen** Congestion.	**Heart** Macroscopic findings: - Areas of green discolouration of the left ventricle, with green, dry, and sandy cut surface; - Green, hard, narrow-banded streaks of the right ventricle. Histologic findings: - Widespread intra- and extracellular Ca^2+^ deposits; - Multiple ossification foci; - Interstitial perimysial fibrosis; - Myocytolytic myocytes mixed with inflammatory cells.		**Heart** Macroscopic findings: - Brown-greenish formation of increased consistency of the right atrial wall. Histologic findings: - Subendocardial calcification surrounded by fibrotic tissue and fibrin deposition; - Necrotic cells; - Interstitial fibrosis; - Inflammatory infiltrate (mainly lymphocytes). **Lungs** - Increased weight and consistency; - Consolidation areas; - Pneumonia.
**Reference**	**de Matos Soeiro et al. [14]** **(2008)** **Retrospective Study**	**Suzuki et al. [15]** **(2009)** **Case Report**		**Aslan et al. [16]** **(2018)** **Case Control Study**
**Findings**	**Lungs** Diffuse Alveolar Damage (DAD – *n* = 1374/3030): - Alveolar collapse; - Hyaline membranes; - Obliterative fibrosis; - Septa neoformation.	**Serous Cavities** Pleural effusion. **Vessels** Pneumohaemia of inner jugular vein, superior vena cava, and cardiac veins. **Gastrointestinal Tract** Macroscopic findings: - Discontinuous areas of oedematous, dark-brown mucosal lesions from terminal ileum to sigmoid colon; - Multiple thrombi of the branches of marginal arteries. Histologic findings: - Gas cysts with lymphoid cells infiltration within colonic submucosa; - Coagulation necrosis involving mucosa, submucosa, and part of the muscular layer; - Areas of ulcer and full-thickness necrosis; - Intestinal transmural infiltration of Gram^-^ rods.		**Kidneys** Glomeruli: - Rare sclerotic changes; - Significant neutrophils and macrophages infiltrates; - Fibrin thrombi. Tubulo-interstitium: - Interstitial inflammation (*n* = 2/27); - Fibrosis (*n* = 7/27); - Tubular atrophy (*n* = 8/27); - Discontinuous acute tubular necrosis (*n* = 24/27); - Significant neutrophils infiltrate; - Apoptotic cells (*n* = 23/27). Peritubular capillaries: - Significant neutrophils infiltrate; - Fibrin thrombi (*n* = 16/27). Other findings: - Vascular intima sclerosis (*n* = 20/27); - Arteriolar hyaline formations (*n* = 14/27).
**Reference**	**Garofalo et al.** [17] **(2019)** **Review**	**Gunia et al. [18]** **(2005)** **Case Control Study**	**Abernathy et al. [19]** **(2019)** **Case Report**	**Gioia et al. [20]** **(2016)** **Case Report**
**Findings**	**Kidneys and Urinary Tract** - Tubulointerstitial nephropathy (tubular cells vacuolization, swelling, brush border injury – *n* = 996/1330); - Acute tubular necrosis (*n* = 236/1330); - Glomerulonephritis (*n* = 34/1330); - Vascular injury (*n* = 13/1330); - Pyelonephritis (*n* = 2/1330). **Brain** - Purpura and white matter haemorrhages (*n* = 17/44); - Microabscesses (*n* = 10/39); - Ischaemic changes (*n* = 25/35); - Rare hypercoagulability, central pontine myelinolysis, and multifocal necrotizing leukoencephalopathy. **Liver** - Intrahepatic cholestasis; - Focal liver cell necrosis; - Hepatocellular apoptosis; - Kupffer cell hyperplasia; - Lobular inflammation; - Portal inflammation; - Cholangiolarbile retention; - Inspissated concretions within dilated cholangioles; - Acute cholangiolitis.	**Spleen** - Lymphoid depletion of B- and T-areas; - Germinal centre hyperplasia.	**External Inspection** Multiple petechiae. **Serous Cavities** Cloudy yellow pericardial fluid. **Heart** Macroscopic findings: - Pericardial erythematous fibrinous debris. Histologic findings: - Coccoid-appearing bacteria surrounded by necrosis and haemorrhage deeply invading myocardial tissue. **Brain** Macroscopic findings: - Grey and white matter, brainstem, and cerebellum haemorrhage. Histologic findings: - Acute inflammation; - Clusters of coccoid-appearing bacteria. **Kidneys** Purulent and haemorrhagic lesions of the renal capsule.	**External Inspection** - Diffuse red-coloured skin; - Cutaneous cellulitis and palpable crepitation; - Oedema of the lymphatic vessels along with papillary dermis expansion. **Abdominal Cavity** - Retroperitoneum gas gangrene, expanding to thigh muscles; - Iliopsoas myonecrosis (brownish discoloration, soft consistency, palpable crepitation); - Mesentery thickening; - Haemorrhagic-oedematous adipose tissues. **Lungs** Macroscopic findings: - Oedema; - Pleural effusion. Histologic findings - Macrophages with brownish granules inside; - Rod-shaped bacteria; - Pulmonary oedema and emphysema. **Kidneys** Myoglobin detection within renal tubules.

**Table 2:** Works discussing the *post-mortem* role of cultural investigations for the aetiological diagnosis of sepsis.

Reference	Cultural Matrix	Microbial Isolate	Diagnosis
**Yang et al.** [8] **(2009)** **Case Series**	Obtained post mortem: Heart, liver, kidneys, cardiac blood	*Streptococcus suis* type 2	Streptococcal Toxic Shock Syndrome (STSS)
**Sledzinska et al.** [10] **(2010)** **Case Report**	Obtained post mortem: Blood, kidney, lung, brain, spleen urine	*Escherichia coli* Dr ^+^	Gestational interstitial tubulonephritis and chronic pyelitis
**Suzuki et al.** [15] **(2009)** **Case Report**	Obtained post mortem: Blood	*Clostridium spp.* *Bacteroide fragilis*	Anaerobic bacterial sepsis due to Pneumatosis Cystoides Intestinalis (PCI)
**Abernathy et al.** [19] **(2019)** **Case Report**	Obtained ante mortem: Blood Obtained post mortem: Pericardial effusion, i. v. tubing, bag of saline	Meticillin-Resistant *Staphylococcus aureus* (MRSA) Meticillin-Resistant *Staphylococcus aureus* (MRSA)	Sepsis following clandestine intravenous infusion
**Gioia et al.** [20] **(2016)** **Case Report**	Obtained post mortem: Aortic blood	*Clostridium perfringens*	Gas gangrene following polypectomy
**Ploya et al.** [22] **(2005)** **Case Report**	Obtained post mortem: Blood, urine, CSF, throat swabs, purpuric lesions, adrenal glands	*Neisseria meningitidis* serogroup C	Purpura fulminans
**Kawaguchi et al.** [23] **(2013)** **Case Report**	Obtained post mortem: Blood, faeces samples	*Streptococcus agalactiae*	Late onset group B streptococcal sepsis
**Frati et al. [24]** **(2015)** **Case Report**	Obtained post mortem: Blood samples	*Y. enterocolitica*	Post-transfusion sepsis
**Sarvari et al. [25]** **(2016)** **Case Report**	Obtained post mortem: Intestinal and subcutaneous tissues	*Clostridium perfringens*	Gas gangrene
**D’Ovidio et al.** [26] **(2015)** **Case Report**	Obtained post mortem: Cardiac tissue and valves, liver, CSF, blood	MDR*K. Pneumoniae*	Sepsis following an incision of a gluteal abscess

CSF: Cerebro-Spinal Fluid; MDR: Multi-Drug Resistant.

**Table 3:** Molecules assessed as potential *post-mortem* immunohistochemical markers of sepsis.

**Reference**	**Tsokos et al.** [30] **(2000)** **Prospective** **Case Control Study**	**Tsokos et al.** [31] **(2001)** **Prospective** **Case Control Study**	**Tsokos et al.** [32] **(2001)** **Prospective** **Case Control Study**	**Tsokos et al.** [33] **(2003)** **Prospective** **Case Control Study**
**Markers** **Assessed**	**E-Selectin** immunoreactivity tested on lung vessels: - Strong in all sepsis cases (mean expression 4.78); - Weak in 29% possible sepsis cases (mean expression 2.35); - Weak in 4% non-sepsis control cases (mean expression 1.5).	Immunoreactivity tested on intravascular, interstitial, and intra-alveolar leucocytes. **VLA-4:** - Strong in all sepsis cases (mean expression 1.8); - Weak or absent in non-sepsis control cases (mean expression 0.1–0.7). **ICAM-1:** - Strong in all sepsis cases (mean expression 2.2); - Weak or absent in non-sepsis control cases (mean expression 0.1).	Immunoreactivity tested on pulmonary leucocytes, and macrophages. **Lactoferrin (LF):** - Strong in all sepsis cases (mean expression 9.0); - Weak in non-sepsis control cases (mean expression 4.5). **Lysozyme (LZ):** - No immunoreactivity difference between sepsis (mean expression 8.8) and non-sepsis control cases (mean expression 6.8).	**VEGF** immunoreactivity tested on alveolar and bronchial epithelium, and glandular cells of the bronchi and bronchioles: - Weak or absent in sepsis cases; - Strong in all non-sepsis control cases.
**Reference**	**Miyashita et al.** [34] **(2006)** **Prospective** **Case Control Study**	**Muller et al.** [35] **(2008)** **Prospective** **Case Control Study**	**Hervig et al.** [36] **(2013)** **Prospective** **Case Control Study**	**An et al.** [37] **(2009)** **Prospective** **Case Control Study**
**Markers** **Assessed**	**TNF-α** immunoreactivity tested on lung macrophages: - Strong in sepsis cases (25% positive ratio); - Weak in non-sepsis cases (13% positive ratio).	Immunoreactivity tested on lung vessels. **VE-Cadherin:** - Reduced in sepsis compared to non-sepsis control cases. **ACE:** - Reduced in sepsis compared to non-sepsis control cases.	**VE-Cadherin** immunoreactivity tested on lung vessels: - Reduced in sepsis compared to non-sepsis control cases.	Immunoreactivity tested on pulmonary macrophages. **CCR2:** - Stronger in sepsis (41.6%) compared to non-sepsis control cases (8%). **CX3CR1:** - Stronger in sepsis (36.2%) compared to non-sepsis control cases (9.2%).
**Reference**	**Maiese et al.** [38] **(2017)** **Prospective** **Case Control Study**	**Ventura Spagnolo et al. [39]** **(2018)** **Retrospective** **Case Control Study**	**Galassi et al.** [40] **(2018)** **Retrospective** **Case Control Study**	**Maiese et al.** [41] **(2019)** **Prospective** **Case Control Study**
**Markers** **Assessed**	**PCT** immunoreactivity tested on brain, heart, lung, liver, and kidney samples: - Significant positivity on cells and vessels of all tissues assessed in nearly all sepsis cases; - Absence of any reaction in all tissue samples in non-sepsis control cases.	**Sarcoglycan** sub-complex tested on heart samples: - Decreased immuoreactivity in all sepsis compared to non-sepsis control cases.	Immunoreactivity tested on heart samples. **LF:** - Significant positivity in all sepsis cases; - Weak or absent positivity in non-sepsis control cases. **CD15:** - Significant positivity in all sepsis cases; - Weak or absent positivity in non-sepsis control cases. No immunoreactivity differences between sepsis and non-sepsis control cases for α-actin, fibronectin, MMP-9, caspase-3, ICAM-1.	**TREM-1** tested on brain, heart, lung, liver, and kidney samples: - Significant positivity in all samples from sepsis cases; - Absent positivity in all tissues from non-sepsis control cases.

E-Selectin: Endothelial-Selectin; VLA-4: Very Late Antigen-4; ICAM-1: Intercellular Adhesion Molecule-1; VEGF: Vascular Endothelial Growth Factor. TNF-α: Tumor Necrosis Factor-α; VE-Cadherin: Vascular Endothelial-Cadherin; ACE: Angiotensin Converting Enzyme; CCR2: C-C Motif Chemokine Receptor 2; CX3CR1: C-X3-C Motif Chemokine Receptor 1. PCT: Procalcitonin; LF: Lactoferrin; CD15: Cluster of Differentiation 15; TREM-1: Triggering Receptor Expressed on Myeloid cells-1; MMP-9: Matrix Metallopeptidase-9; ICAM-1: Intercellular Adhesion Molecule-1.

**Table 4:** Molecules assessed as possible post-mortem biochemical markers of sepsis.

**Reference**	**Tsokos et al.** [44] **(2001)** **Prospective** **Case Control Study**	**Reichelt et al.** [45] **(2005)** **Prospective** **Case Control Study**	**Schrag et al. [46]** **(2012)** **Prospective** **Case Control Study**	**Schrag et al.** [47] **(2012)** **Prospective** **Case Control Study**	**Augsburger et al.** [48] **(2013)** **Prospective** **Case Control Study**
**Cut-offs**	**CRP** → 10 mg/L **IL-6** → 10 pg/mL	**sIL-1β** → 5 pg/mL **sIL-2R** → 1.000 U/mL **LBP** → 10 µg/mL	**CRP** → 10 mg/L **PCT**→ 0.25 ng/mL **TNF-α**, **IL-6** and **IL-8** cut-offs not reported	**CRP** → 10 mg/L **PCT** → 2 µg/L	**PCT** → 2 µg/L **LBP** → 10 µg/mL
**Findings**	**CRP** Sepsis cases (n = 8): - Range levels in ante mortem samples = 42–293 mg/L; - Range levels in post mortem samples = 31–309 mg/L, decreasing with PMI progression. Control cases (n = 16): - <10 mg/L in 8/16 cases; - Undetectable in 8/16 cases. **IL-6** Sepsis cases (n = 8): - Range levels in ante mortem samples = 673–29.785 pg/mL; - Range levels in post mortem samples = 739–331.600 pg/mL, increasing with PMI progression in 5/8 cases. Control cases (n = 16): - Range levels in post mortem samples = 5–1.364 pg/mL, increasing with PMI progression in 6/16 cases, without exceeding 1.500 pg/mL.	**sIL-1β** Sepsis cases (n = 8): - Range levels in ante mortem samples = 5.5–11 pg/mL in 3/8 cases; 3.660 in 1/8 cases; - Range levels in post mortem samples = 5.2–229.1 pg/mL in 6/8 cases; 13.685.7pg/mL in 1/8 cases. Increasing levels with PMI progression. Control cases (n = 16): - Range levels in post mortem samples = 5–231.3 pg/mL, increasing with PMI progression in 11/16 cases. **sIL-2R** Sepsis cases (n = 8): - Range levels in ante mortem samples = 2.514–12.462 U/mL; - Range levels in post mortem samples = 1.274.6–10.809.7 U/mL, decreasing with PMI progression. Control cases (n = 16): - Range levels in post mortem samples = 230–1.008.3 U/mL, decreasing with PMI progression. **LBP** Sepsis cases (n = 8): - Range levels in post mortem samples = 99.1–15.2 µg/mL, decreasing with PMI progression. Control cases (n = 16): - Range levels in post mortem samples = 13.1–3.8 µg/mL, decreasing with PMI progression.	**CRP** Sepsis cases (n = 8): - Range levels detected = 23–272 mg/L; Control cases (n = 10): - <15 mg/L in 9/10 cases. **PCT** Sepsis cases (n = 8): - Range levels detected = 0.03–218 ng/mL in 7/8 cases; Control cases (n = 10): - <0.5 ng/mL in 9/10 cases. **TNF-α** Sepsis cases (n = 8): - Range levels detected = 2.92–249 pg/mL in 6/8 cases; Control cases (n = 10): - Range levels detected = 0.1–586 pg/mL. **IL-6** Sepsis cases (n = 8): - Range levels detected = 81–75.296 pg/mL; Control cases (n = 10): - Range levels detected = 16–112.404 pg/mL. **IL-8** Sepsis cases (n = 8): - Range levels detected = 79–3.115 pg/mL; Control cases (n = 10): - Range levels detected = 0–399 pg/mL.	**CRP** Sepsis cases (n = 12): - Range levels in serum = 19–285 mg/L in 11/12 cases; - Range levels in pericardial fluid = 19–87 mg/L in 9/12 cases. Control cases (n = 28): - Undetectable, both in serum and pericardial fluid, in 25/28 cases. **PCT** Sepsis cases (n = 12): - Range levels in serum = 3.68–85.50 µg/L; - Range levels in pericardial fluid = 5.40–131.101 µg/L in 11/12 cases. Control cases (n = 28): - <2 µg/L in 3/28 cases in serum samples; - Undetectable, both in serum and pericardial fluid, in 25/28 cases.	**PCT** Sepsis cases (n = 12): - Range in serum samples = 2.31–155.70 µg/L in 11/12 cases; - Range in pericardial fluid samples = 2.30–80.15 µg/L, in 9/12 cases. Control cases (n = 30): - <2 µg/L in 28/30 cases, both in serum and pericardial fluid. **LBP** Sepsis cases (n = 12): - Range in serum samples = 15.4–70.1 µg/mL in 11/12 cases; - Range in pericardial fluid samples = 8.1–10.2 µg/mL, in 2/12 cases. Control cases (n = 30): - <10 µg/mL in 29/30 cases in serum samples; - <10 µg/mL in 28/30 cases in pericardial fluid samples.
**Reference**	**Palmiere et al.** [49] **(2013)** **Prospective** **Case Control Study**	**Palmiere et al.** [50] **(2013)** **Prospective** **Case Control Study**	**Palmiere et al.** [51] **(2014)** **Prospective** **Case Control Study**	**Palmiere et al.** [52] **(2014)** **Prospective** **Case Control Study**	
**Cut-offs**	**CRP** → 10 mg/L **PCT** → 2 µg/L **sTREM-1** → 90 pg/mL	**CRP** → 10 mg/L **PCT** → 2 µg/L **sCD14-ST** → 600 pg/mL	**CRP** → 10 mg/L **PCT** → 2 µg/L **sTREM-1** → 90 pg/mL **sIL-2R** → 5 ng/mL	**CRP** → 10 mg/L **PCT** → 2 µg/L **IL-6** → 200 pg/mL **Copeptin** → 15.6 pg/mL	
**Findings**	**CRP** Sepsis cases (n = 16): - Range in serum samples = 56–372 mg/L in 15/16 cases. Control cases (n = 16): - < 10 mg/L in 13/16 cases. **PCT** Sepsis cases (n = 16): - Range in serum samples = 2.50–155.70 µg/L in 15/16 cases. Control cases (n = 16): - < 2 µg/L in 13/16 cases. sTREM-1 Sepsis cases (n = 16): - Range in serum samples = 95–280 pg/mL in 14/16 cases. Control cases (n = 16): - <90 pg/mL in 12/16 cases.	**CRP** Sepsis cases (n = 19): - Range in serum samples = 25–372 mg/L in 18/19 cases. Control cases (n = 18): - <10 mg/L in 14/18 cases. **PCT** Sepsis cases (n = 19): - Range in serum samples = 2.31–155.70 µg/L in 18/19 cases. Control cases (n = 18): - <2 µg/L in 15/18 cases. **sCD14-ST** Sepsis cases (n = 19): - Range in serum samples = 750–8.960 pg/mL in 18/19 cases; Control cases (n = 18): - <600 pg/mL in 8/18 cases.	**CRP** Sepsis cases (n = 12): - Range levels in serum samples = 21–134 mg/L; - Range levels in pericardial fluid samples = 28–160 mg/L; - Range levels in pleural fluid samples = 40–144 mg/L. **PCT** Sepsis cases (n = 12): - Range levels in serum samples = 2.12–7.59 µg/L; - Range levels in pericardial fluid samples = 2.24–7.84 µg/L; - Range levels in pleural fluid samples = 2.40–8.65 µg/L. **sTREM-1** Sepsis cases (n = 12): - Range levels in serum samples = 150–300 pg/mL in 11/12 cases; - Range levels in pericardial fluid samples = 150–420 pg/mL in 11/12 cases; - Range levels in pleural fluid samples = 130–460 pg/mL in 11/12 cases. **sIL-2R** Sepsis cases (n = 12): - Range levels in serum samples = 6–9 ng/mL in 9/12 cases; - Range levels in pericardial fluid samples = 6–9 ng/mL in 9/12 cases; - Range levels in pleural fluid samples = 6–9 ng/mL in 7/12 cases.	**CRP** Sepsis cases (n = 28): - Range levels detected = 38–285 mg/L. Control cases (n = 28): - Range levels detected = 2–40 mg/L. **PCT** Sepsis cases (n = 28): - Range levels detected = 2.14–6.06 µg/L. Control cases (n = 28): - Range levels detected = 0.06–0.16 µg/L. **IL-6** Sepsis cases (n = 28): - Range levels detected = 400–29.180 pg/mL. Control cases (n = 28): - Range levels detected = 25–180 pg/mL. **Copeptin** Sepsis cases (n = 28): - Range levels detected = 104–341 pg/mL. Control cases (n = 28): - Range levels detected = 16–96 pg/mL.	
**Reference**	**Palmiere et al.** [53] **(2014)** **Prospective** **Case Control Study**	**Palmiere et al.** [54] **(2015)** **Prospective** **Case Control Study**	**Tettamanti et al.** [55] **(2016)** **Retrospective** **Case Control Study**	**Unuma et al.** [56] **(2019)** **Retrospective** **Case Control Study**	
**Cut-offs**	**CRP** → 10 mg/L **PCT** → 2 µg/L **Endocan** → 1.200 ng/mL	**CRP** → 10 mg/L **PCT** → 2 µg/L **sTREM-1** → 90 pg/mL **IL-6** → 200 pg/mL **Psp/reg** → 1 ng/mL	**Troponin I** → 0.03 µg/L **Troponin T** → 14 ng/L **NT-proBNP** → 738 pg/mL	**CRP** → 7 mg/dL **PCT** → 0.07 ng/mL **PSEP** → 1250 pg/mL	
**Findings**	**CRP** Sepsis cases (*n* = 16): - Range levels in serum samples = 47–209 mg/L in 15/16 cases. Control cases (*n* = 16): - <10 mg/L in 13/16 cases. **PCT** Sepsis cases (*n* = 16): - Range levels in serum samples = 2.89–11.0 4 µg/L in 15/16 case. Control cases (*n* = 16): - <2 µg/L in 14/16 cases in serum samples. **Endocan** Sepsis cases (*n* = 16): - Range levels in serum samples = 1.409–6.756 ng/mL in 15/16 cases; - <1.200 ng/mL in 14/16 cases in pericardial fluid samples. Control cases (*n* = 16): - <1.200 ng/mL in 15/16 cases in serum samples; - <1.200 ng/mL in 14/16 cases in pericardial fluid samples.	**CRP** Sepsis cases (*n* = 20): - Range levels detected = 45–140 mg/L. Control cases (*n* = 20): - Range levels detected = 4–35 mg/L. **PCT** Sepsis cases (*n* = 20): - Range levels detected = 1.68–5.66 µg/L. Control cases (*n* = 20): - Range levels detected = 0.06–0.14 µg/L. **sTREM-1** Sepsis cases (*n* = 20): - Range levels detected = 58–260 pg/mL. Control cases (*n* = 20): - Range levels detected = 23–97 pg/mL. **IL-6** Sepsis cases (*n* = 20): - Range levels detected = 680–3840 pg/mL. Control cases (*n* = 20): - Range levels detected = 25–350 pg/mL. **Psp/reg** Sepsis cases (*n* = 20): - Range levels detected = 0.8–2.9 ng/mL. Control cases (*n* = 20): - Range levels detected = 0.1–1.1 ng/mL.	**Troponin I** Sepsis cases (*n* = 16): - Range levels detected = 0.99–9.16 µg/L. Control cases (*n* = 16): - <0.03 µg/L in 14/16 cases. **Troponin T** Sepsis cases (*n* = 16): - Range levels detected = 21–95 ng/L. Control cases (*n* = 16): - <14 ng/L in 14/16 cases. **NT-proBNP** Sepsis cases (*n* = 16): - Range levels detected = 2.678–10.680 pg/mL. Control cases (*n* = 16): - <738 pg/mL in 15/16 cases.	**CRP** Sepsis cases (*n* = 19): - Range levels detected = 7.27–48.60 mg/dL in 15/19 cases (mean 17.33 ± 2.09). Control cases (*n* = 74): - Mean level detected = 5.12 ± 1.06 mg/dL. **PCT** Sepsis cases (*n* = 19): - Range levels detected = 0.07–77.40 ng/mL in 16/19 cases (mean 5.303 ± 1.818). Control cases (*n* = 74): - Mean level detected = 0.407–0.921 ng/mL. **PSEP** Sepsis cases (*n* = 19): - Range levels detected = 1.320–11.000 pg/mL (mean 3.253.2 ± 279.1). Control cases (*n* = 28): - Mean level detected = 562.5–141.4 pg/mL.	

PMI: Post mortem Interval; PCT: Procalcitonin; CRP: C-Reactive Protein; IL-6: Interleukin-6; IL-8: Interleukin-8; sIL-1β: soluble Interleukin-1β; sIL-2R: soluble Interleukin-2 Receptor; LBP: Lipopolysaccharide Binding Protein; TNF-α: Tumor Necrosis Factor-α; sTREM-1: soluble Triggering Receptor Expressed on Myeloid cells-1; sCD14-ST: soluble Cluster of Differentiation 14-Subtype; sIL-2R; Psp/reg: Pancreatic stone protein/regenerating protein; PSEP: Presepsin.
